# K^+^ homeostasis is important for survival of *Acinetobacter baumannii* ATCC 19606 in the nosocomial environment

**DOI:** 10.1007/s10123-023-00389-3

**Published:** 2023-06-20

**Authors:** Patricia König, Beate Averhoff, Volker Müller

**Affiliations:** Department of Molecular Microbiology & Bioenergetics, Institute of Molecular Biosciences, Goethe-University, Max-von-Laue-Str. 9, 60438 Frankfurt am Main, Germany

**Keywords:** Pathogen, Osmostress, Virulence, Persistence, Drought

## Abstract

Pathogenic bacteria have developed several mechanisms to thrive within the hostile environment of the human host, but it is often disregarded that their survival outside this niche is crucial for their successful transmission. *Acinetobacter baumannii* is very well adapted to both the human host and the hospital environment. The latter is facilitated by multifactorial mechanisms including its outstanding ability to survive on dry surfaces, its high metabolic diversity, and, of course, its remarkable osmotic resistance. As a first response to changing osmolarities, bacteria accumulate K^+^ in high amount to counterbalance the external ionic strength. Here, we addressed whether K^+^ uptake is involved in the challenges imposed by the harsh conditions outside its host and how K^+^ import influences the antibiotic resistance of *A. baumannii*. For this purpose, we used a strain lacking all major K^+^ importer ∆*kup*∆*trk*∆*kdp.* Survival of this mutant was strongly impaired under nutrient limitation in comparison to the wild type. Furthermore, we found that not only the resistance against copper but also against the disinfectant chlorhexidine was reduced in the triple mutant compared to the wild type. Finally, we revealed that the triple mutant is highly susceptible to a broad range of antibiotics and antimicrobial peptides. By studying mutants, in which the K^+^ transporter were deleted individually, we provide evidence that this effect is a consequence of the altered K^+^ uptake machinery. Conclusively, this study provides supporting information on the relevance of K^+^ homeostasis in the adaptation of *A. baumannii* to the nosocomial environment.

## Introduction

The nosocomial pathogen *Acinetobacter baumannii* has become a major threat in healthcare institutions worldwide, initiating global research activities to better understand the general physiology of this Gram-negative bacterium and the mechanisms of infections and persistence in the hospital environment. One outstanding feature of *A. baumannii* is its extremely high desiccation resistance, which is strain specific (Wendt et al. 1997; Jawad et al. 1998; Espinal et al. 2012; Zeidler and Müller 2019). Another important trait of *A.* *baumannii* is its ability to withstand a broad range of different osmolarities, enabling it to colonize different areas within the human host (Dijkshoorn et al. 2007; König et al. 2021). In an ongoing effort to decipher the molecular basis for desiccation and osmostress resistance, we recently identified CsrA as a key player in compatible solute synthesis (Hubloher et al. 2021). CsrA was found to act either as an activator or repressor for the synthesis of glutamate and mannitol in a strain-dependent manner. A deletion of *csrA* increased osmostress resistance in *A. baumannii* 17961 and AB09-003 while not affecting it in *A. baumannii* ATCC 19606 (Farrow et al. 2020; Hubloher et al. 2021).

The physiological response to low water activities by *A. baumannii* is the accumulation of compatible solutes to prevent loss of intracellular water (Zeidler et al. 2017, 2018; Zeidler and Müller 2019). In earlier studies, we deciphered the role of K^+^ in the early stage of osmostress response and compatible solute synthesis (König et al. 2020). *A. baumannii* has three major K^+^ transporter, the primary transporter Kdp that is active under low K^+^ concentrations to ensure K^+^ accumulation by ATP hydrolysis even at low external K^+^ and two secondary K^+^ transporter driven by the electrochemical ion potential across the membrane, Kup and Trk, operating with low affinity but high capacity under high K^+^ concentrations (Samir et al. 2016; König et al. 2021). Genetic deletions of the transporter underscored the importance of K^+^ uptake in sensing osmolarity and surviving in high-osmolarity environments such as human urine, one of the ecological niches for *A. baumannii* (König et al. 2021). Here, we have addressed the role of the major K^+^ transporter of *A. baumannii* in cellular processes involved in persistence and resistance. Therefore, we used a mutant devoid of all these K^+^ transporter and imposed different stresses mimicking challenges in the hospital environment. These experiments revealed a role of K^+^ in survival under ambient temperatures and resistance to disinfectants and antibiotics as well as metal stress resistance.

## Materials and methods

### Bacterial strains and culture conditions

Cells of *A. baumannii* ATCC 19606^ T^ and the ∆*kup*, ∆*trk*, ∆*kdp*, ∆*kup*∆*trk*, and ∆*kup*∆*trk*∆*kdp* deletion mutants (König et al. 2021) were mainly grown in minimal medium with 20 mM succinate as sole carbon and energy source (Zeidler et al. 2017). Occasionally and as specified in the text, complex medium (LB) (Bertani 1951) was used. Growth conditions and medium preparation were as described before (Zeidler et al. 2017).

## Determination of intracellular ATP content

Cells of the wild type and the triple mutant were grown overnight in minimal medium with 20 mM succinate as carbon source. The culture was centrifuged and washed twice in sterile phosphate buffered saline (PBS, 140 mM NaCl, 10 mM KCl, 16 mM Na_2_HPO_4_, 2 mM KH_2_PO_4_) prior to the determination of the protein content of the cell suspension by Schmidt et al. (1963). Ten milliliters of a 1-mg protein/ml cell suspension was preincubated at 37 °C prior to the addition of 12 mM succinate to initiate ATP synthesis. At indicated time points, samples of 400 μl were collected and intracellular ATP content was determined as described (Breisch and Averhoff 2020).

## Determination of chlorhexidine resistance by disc diffusion assay

The effect of chlorhexidine on the survival of *A. baumannii* strains was studied by a disc diffusion test. Therefore, cells were grown to late stationary growth phase (here: 2 h after reaching stationary growth phase) in minimal medium (26.5 mM K^+^, 20 mM succinate), washed thrice in sterile saline (0.9% NaCl), and adjusted to OD_600nm_ = 1. One hundred fifty microliters of this suspension was plated on LB agar plates before a 9-mm filter disc soaked in 0.8% chlorhexidine was placed in the middle of the plate. Plates were incubated overnight at 37 °C and the diameter of the inhibition zone was measured.

## Drop dilution assay

To study the effect of different antibacterial agents, drop dilution assays were performed. Therefore, cells were cultivated in minimal medium (26.5 mM K^+^, 20 mM succinate) to stationary growth phase, washed thrice in sterile saline, and adjusted to OD_600nm_ = 1. Ten microliters of serial dilutions were spotted on LB agar plates containing antibiotics. Plates were incubated overnight at 37 °C. To test the effect of K^+^ limitation, serial dilutions were spotted on minimal medium agar plates containing either 26.5 mM K^+^ or no additional K^+^ (trace amounts are left).

## Long-term survival

For the long-term survival assay cells were cultivated in minimal medium containing 20 mM succinate at 22 °C to stationary growth phase. Cells were centrifuged, washed thrice in sterile saline, and adjusted to OD_600nm_ = 2. Cells were incubated on a rotary shaker at 22 °C. Survival was monitored by determining CFU (colony forming units) at time points indicated.

## Statistical analysis

Graphs were generated using GraphPad Prism 6 software. Each experiment was performed at least three times and the standard deviation from the mean was calculated. For all statistical analyses, GraphPad Prism software was used. Standard deviations were analyzed using Student’s *t* test. Differences were considered statistically significant when *p* ≤ 0.05.

## Results

### K^+^ transporter deletion mutants are defective in growth and survival at room temperature

**Fig. 1:**
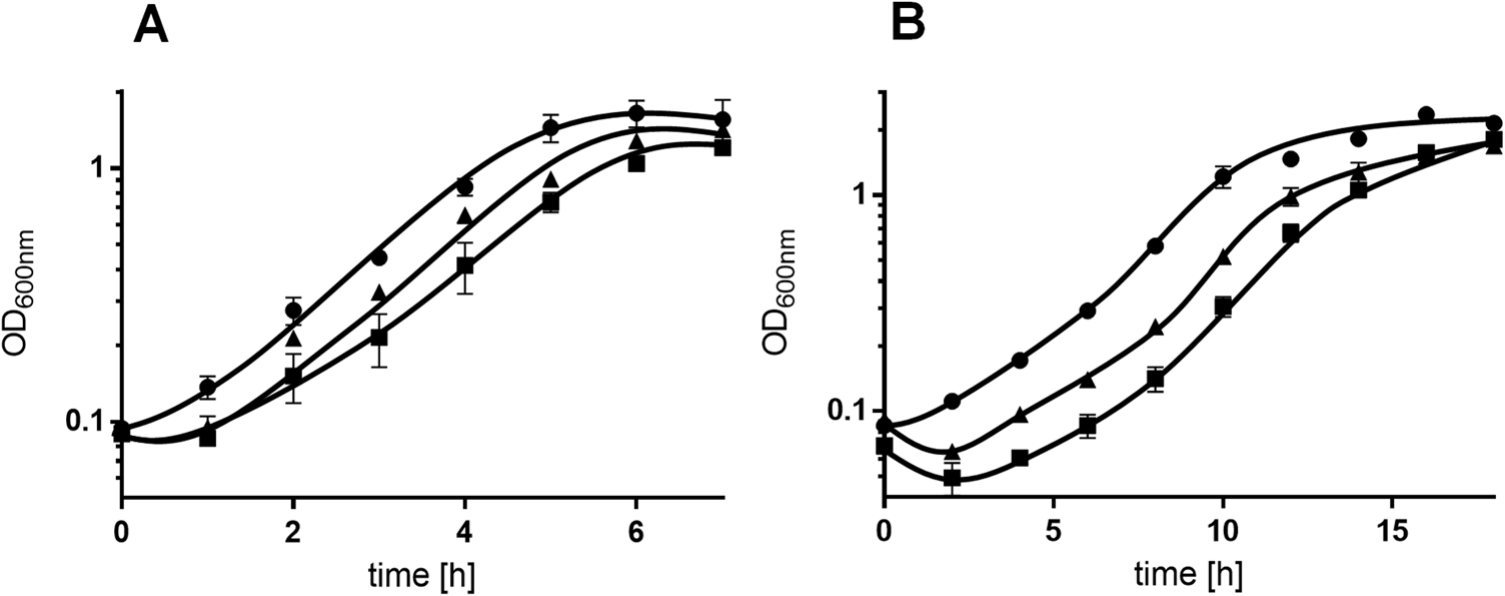
Deletion of K^+^ transporter genes affects growth at room temperature. Cells of the wild type (●), ∆*kup*∆*trk* (▲), and ∆*kup*∆*trk*∆*kdp* (■) were cultivated in minimal medium containing 20 mM succinate and were then transferred to fresh medium (26.5 mM K.^+^, 20 mM succinate). Growth was observed by determining optical density at 600 nm. Cultures were incubated at either 37 °C (**A**) or 22 °C (**B**). Error bars indicate the standard deviation from at least three biological replicates and were analyzed with Student’s *t* test. Differences were considered statistically significant when *p* ≤ 0.05

The nosocomial environment is one of the postulated ecological niches for *A. baumannii* (Dijkshoorn et al. 2007). The optimal growth temperature of *A. baumannii* is 37 °C and, therefore, one essential aspect of survival in the hospital environment is to cope with room temperature (22 °C). To elucidate the role of K^+^ under these conditions, the growth of the K^+^ transporter deletion mutants was analyzed. At 37 °C growth of the double and triple mutant was only slightly affected (*p* = 0.0269 (double mutant), *p* = 0.0109 (triple mutant); Fig. 1A). Here, the lag phase of the double and triple mutant was 2 h instead of 1 h compared to the wild type. Lowering the temperature from 37 to 22 °C reduced the growth rate of the wild type by 35% (37 °C: 0.64 h^−1^; 22 °C: 0.42 ± 0.04 h^−1^) (Fig. 1B). The double mutant ∆*kup*∆*trk* and the triple mutant ∆*kup*∆*trk*∆*kdp* had identical growth rates and grew to identical optical densities but showed increased lag phases in comparison to the wild type. The double mutant had a lag phase of 4 h, whereas the triple mutant had an even longer lag phase of about 6 h (Fig. 1). Both growth defects were significantly different from wild type growth (*p* = 0.0035 (double mutant), *p* = 0.0028 (triple mutant)).

**Fig. 2:**
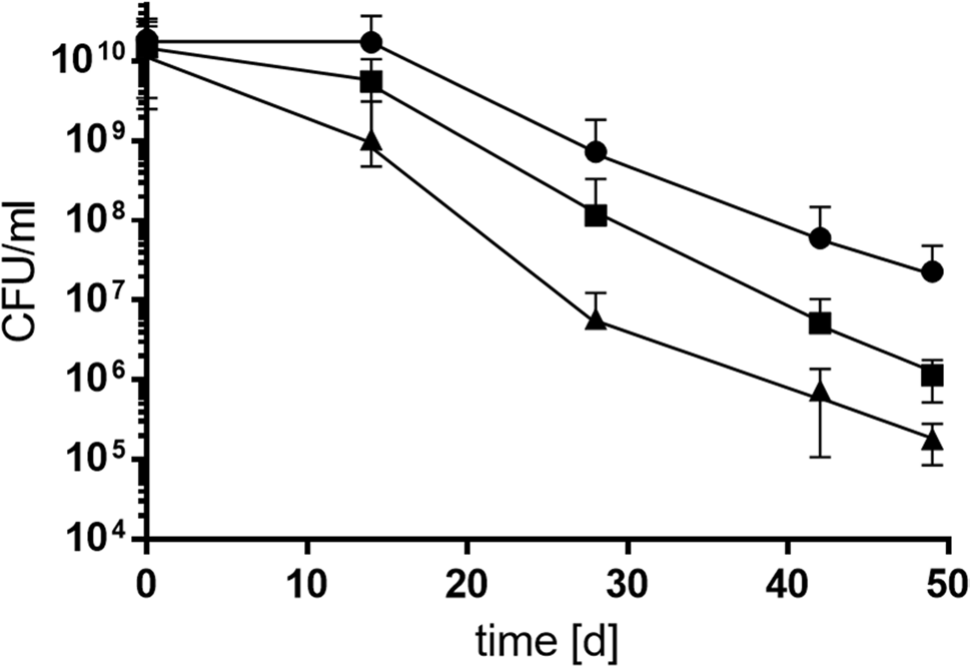
K^+^ uptake is important for long-term survival at room temperature. Cells of the wild type (●), ∆*kup*∆*trk* (■), and ∆*kup*∆*trk*∆*kdp* (▲) were cultivated in minimal medium containing 20 mM succinate at 37 °C. Stationary cultures were centrifuged, washed, and adjusted to OD_600nm_ = 2 in sterile saline (0.9% NaCl). Samples were incubated on a rotary shaker for 50 days at 22 °C. CFU/ml were determined at time points indicated. Error bars indicate the standard deviation from at least three biological replicates and were analyzed with Student’s *t* test. Differences were considered statistically significant when *p* ≤ 0.05

The key to successful transmission inside the hospital is to persist under harsh conditions for long periods of time. Therefore, we analyzed survival of cells in solution at 22 °C. To this end, the wild type, the double mutant, and the triple mutant were grown to stationary phase in minimal medium containing 20 mM succinate as sole carbon and energy source, harvested, and washed three times in sterile saline. Cells were then resuspended again, adjusted to an OD = 2, and incubated for 50 days on a rotary shaker at 22 °C in sterile saline. The viability of the wild type decreased gradually over time with a rate (μ_D_) of 0.2 ± 0.05 d^−1^ (increase of dead cells per total cell count per day) (Fig. 2). The dying rate of the double mutant increased by 24% although not significantly (0.26 ± 0.04 d^−1^; *p* = 0.2235) and for the triple mutant an even faster, significant loss of viability could be observed (0.31 ± 0.04 d^−1^; *p* = 0.0388). After 50 days, the viable counts were reduced to 1 × 10^6^ CFU/ml in the double and 1 × 10^5^ CFU/ml in the triple mutant, whereas viability was still high in the wild type with 10^8^ CFU/ml.

### The ∆*kup*∆*trk*∆*kdp* mutant is impaired in copper resistance and in growth under iron limitation

**Fig. 3:**
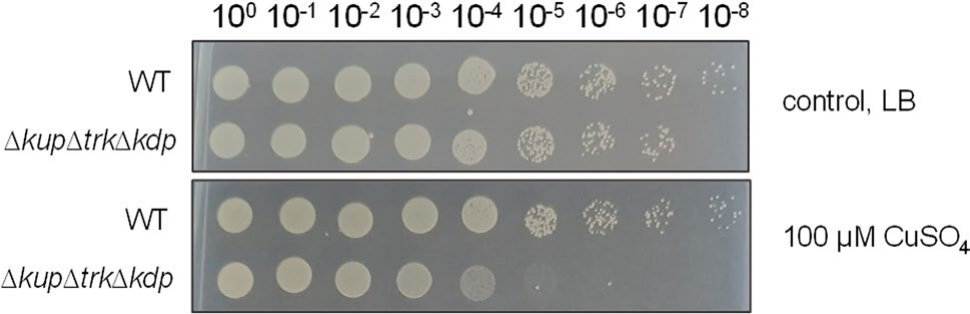
Loss of efficient K^+^ transport reduces copper resistance. Cells of the wild type and the triple mutant were grown overnight in minimal medium (26.5 mM K^+^, 20 mM succinate), washed three times, and adjusted to OD_600nm_ = 1 in sterile saline (0.9% NaCl). Ten microliters of serial dilutions were spotted on LB agar plates containing 100 μM CuSO_4_. Plates were incubated overnight at 37 °C. One representative experiment of at least three biological replicates is shown

Medical devices like ventilators or catheters are coated with antibacterial alloys, e.g., copper, to reduce infections. Therefore, we tested the role of K^+^ in copper resistance via a drop dilution assay. One hundred micromolar of CuSO_4_ was added to LB agar plates and serial dilutions of the wild type and the triple mutant were spotted on the plates (Fig. 3). While growth of the wild type was unaffected by the presence of 100 μM CuSO_4_, growth of the triple mutant was reduced drastically. The growth of the double and single mutants was not affected by 100 μM CuSO_4_ (data not shown).

**Fig. 4:**
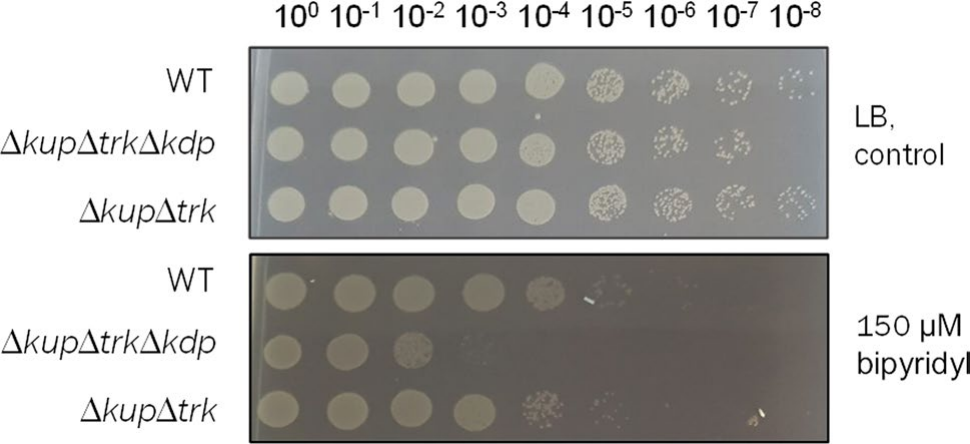
Growth under iron limitation is impaired in the triple mutant. Cells of the wild type, double mutant, and triple mutant were grown in minimal medium (26.5 mM K^+^, 20 mM succinate) to stationary growth phase, washed thrice, and adjusted to OD_600nm_ = 1 in sterile saline (0.9% NaCl). Ten microliters of serial dilutions were spotted on LB agar plates containing 150 μM 2,2ʹ-bipyridyl, an iron chelating molecule. Plates were incubated overnight at 37 °C. One representative experiment of at least three biological replicates is shown

Like copper, iron is essential for microbial life and the availability of soluble iron (Fe^2+^) is limited under aerobic conditions. To analyze the effect of K^+^ on iron acquisition and utilization, a drop dilution assay was performed. Serial dilutions of the wild type and the double and triple mutant were spotted on LB agar plates supplemented with 150 μM 2,2ʹ-bipyridyl, an iron chelating molecule, to mimic iron limitation (Fig. 4). While the wild type grew unaffected, the growth of the double and triple mutant was reduced up to 4 log-fold. Again, growth of the single mutants was not impaired under these conditions (data not shown).

### The ∆*kup*∆*trk*∆*kdp* mutant is impaired in chlorhexidine resistance

**Fig. 5:**
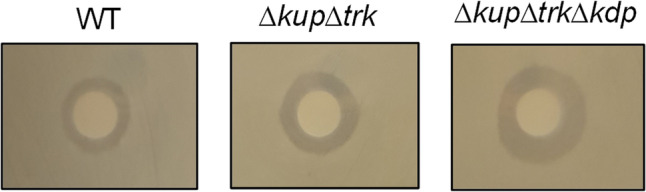
K^+^ uptake contributes to chlorhexidine resistance. Cells of the wild type, double mutant, and triple mutant were grown overnight in minimal medium (26.5 mM K^+^, 20 mM succinate), washed three times in sterile saline (0.9% NaCl), and adjusted to OD_600nm_ = 1 before a disc diffusion assay was performed with 0.8% chlorhexidine. Plates were incubated overnight at 37 °C and the diameter of the inhibition zone was measured. One representative experiment of at least three biological replicates is shown. The standard deviation from at least three biological replicates was analyzed with Student’s *t* test. Differences were considered statistically significant when *p* ≤ 0.05

Over decades, bacteria have established different resistance mechanisms against disinfectants (Mc Carlie et al. 2020; Tong et al. 2021). Here, we analyzed whether K^+^ is involved directly or indirectly in resistance against chlorhexidine with the wild type, double mutant, and triple mutant using chlorhexidine-containing agar plates. The inhibition zone was smallest in the wild type and increased statistically significantly by 24% (1.7 ± 0.2 cm; *p* = 0.0001) in the double mutant. The triple mutant ∆*kup*∆*trk*∆*kdp* was the most sensitive with a significantly increased inhibition zone of 2.1 ± 0.2 cm (*p* = 0.0097; Fig. 5). The growth of the single mutants was as reduced as the one of the wild type (data not shown).

## Role of K^+^ transport in maintaining cellular energy charge

**Fig. 6:**
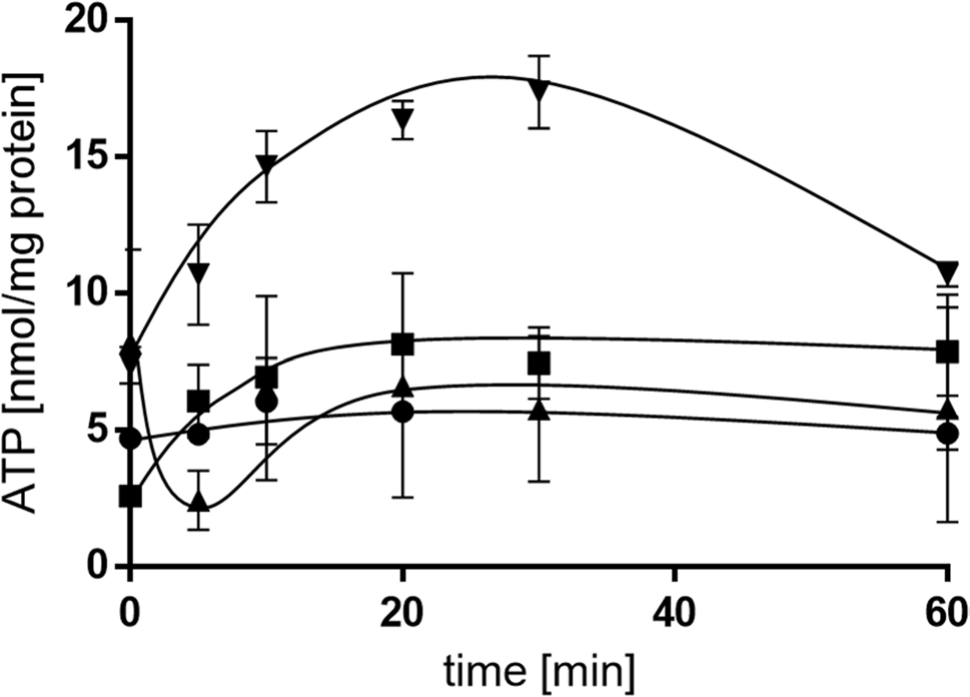
Deletion of K^+^ transporter increases the initial cellular ATP content. Cells of the wild type and the triple mutant were pre-grown in minimal medium containing 20 mM succinate overnight. Cells were harvested, washed in sterile phosphate buffered saline (PBS), and resuspended to a protein concentration of 1 mg/ml in PBS. ATP synthesis in the wild type (■) and the ∆*kup*∆*trk*∆*kdp* strain (▼) was started by the addition of 12 mM succinate. Additional controls without the addition of succinate were performed (wild type: ●; ∆*kup*∆*trk*∆*kdp*: ▲). ATP concentrations were determined at the time points indicated. Error bars indicate the standard deviation from at least three biological replicates and were analyzed with Student’s *t* test. Differences were considered statistically significant when *p* ≤ 0.05

K^+^ is actively taken up by cells, driven by ATP-hydrolysis or the transmembrane electrochemical ion gradient (Epstein 2003; Stautz et al. 2021). To determine the effect of K^+^ on the cellular energy level, cell suspensions of the wild type and the triple mutant in phosphate buffered saline were prepared. In the absence of a substrate, the cellular ATP level stayed constant at around 5 nmol/mg protein (Fig. 6). Upon addition of succinate, the ATP concentration in the wild type increased by about 50% and remained stable at a concentration of 7.5 nmol/mg protein. In contrast, the increase in the ATP content in the triple mutant was significantly more pronounced and reached 17.4 ± 1.3 nmol/mg protein within the first 30 min (*p* = 0.0022). Directly after reaching its maximum, ATP levels decreased to wild type levels again.

## Intracellular K^+^ limitation decreases antibiotic resistance

**Fig. 7:**
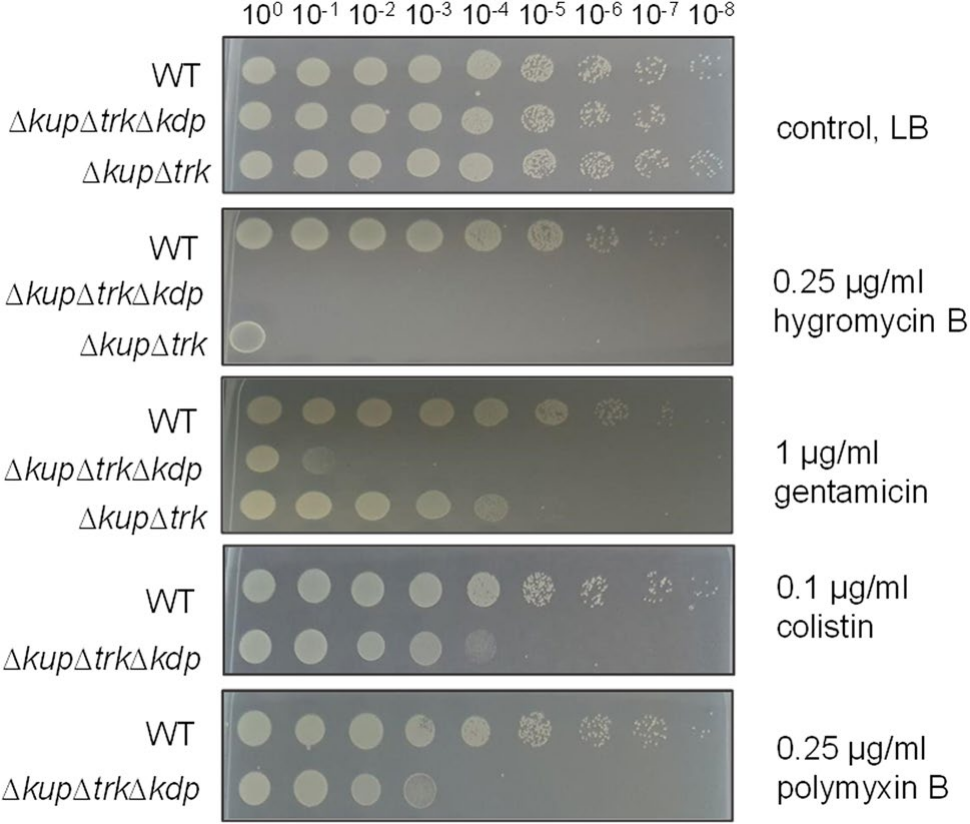
Deletion of K^+^ transporter abolishes antibiotic resistance. Cells of the wild type, double mutant, and triple mutant were grown in minimal medium (26.5 mM K^+^, 20 mM succinate) to stationary growth phase, washed thrice, and adjusted to OD_600nm_ = 1 in sterile saline (0.9% NaCl). Ten microliters of serial dilutions was spotted on LB agar plates containing either 0.25 μg/ml hygromycin B, 1 μg/ml gentamicin, 0.1 μg/ml colistin, or 0.25 μg/ml polymyxin B. LB agar plates without additional antibiotics served as a control. Plates were incubated overnight at 37 °C. One representative experiment of at least three biological replicates is shown

Next, we analyzed the role of K^+^ transport in antibiotic resistance. To this end, we performed a drop dilution assay using either 0.25 μg/ml hygromycin B, 1 μg/ml gentamicin, 0.1 μg/ml colistin, or 0.25 μg/ml polymyxin B. Serial dilutions of the wild type, double mutant, and triple mutant were spotted on antibiotic-containing agar plates (Fig. 7). In the presence of hygromycin B, growth of the triple mutant was completely abolished, while the double mutant grew only in the assay with an undiluted cell suspension. In the presence of gentamicin, the triple and double mutant grew up to a dilution of 10^−1^ and 10^−4^, respectively, whereas the growth of the wild type remained nearly inhibited. Colistin and polymyxin had no effect on the growth of the wild type and the double mutant (data not shown) but inhibited growth of the triple mutant. The growth of the single mutants was unaffected by the concentrations used (data not shown).

**Fig. 8:**
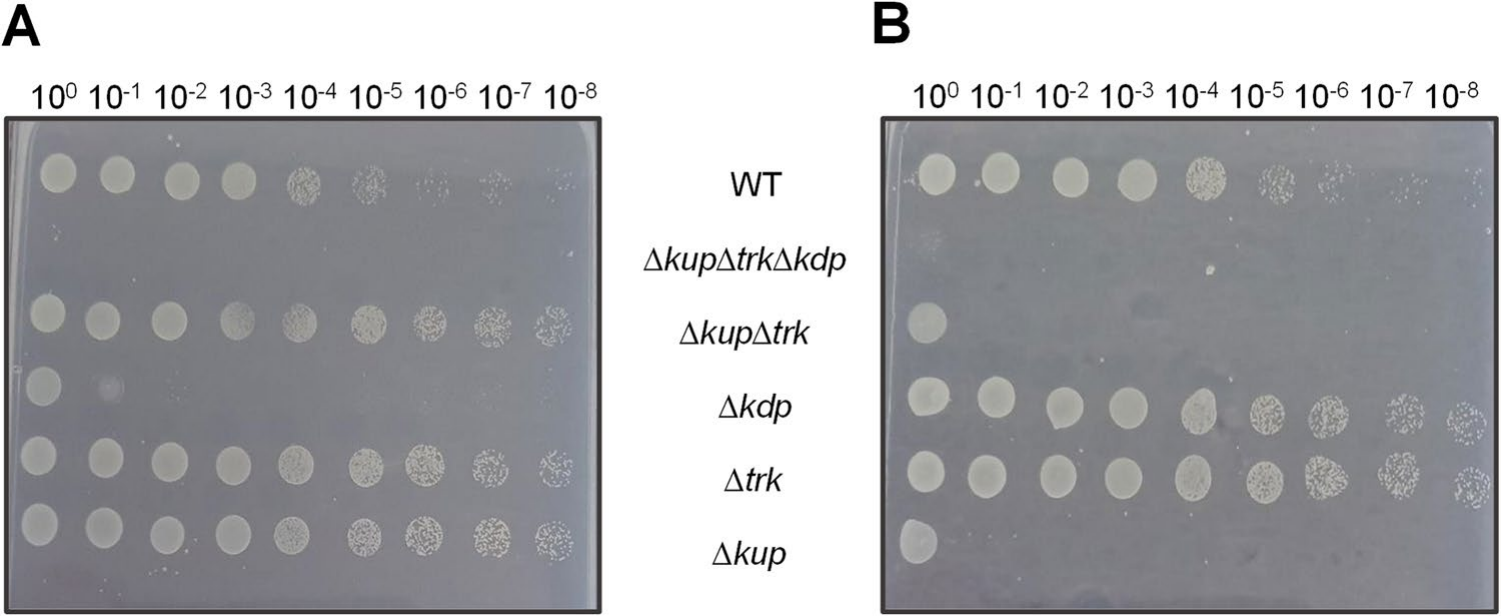
Antibiotic resistance is dependent on K^+^ transporter activity. Cells of the wild type, the single mutants, and the double and triple mutants were grown in minimal medium (26.5 mM K^+^, 20 mM succinate) to stationary growth phase, washed thrice, and adjusted to OD_600nm_ = 1 in sterile saline (0.9% NaCl). Ten microliters of serial dilutions was spotted on minimal medium agar plates with either trace amounts of K^+^ (A) or 26.5 mM K^+^ (B) containing 8 μg/ml gentamicin. Plates were incubated overnight at 37 °C. One representative experiment of at least three biological replicates is shown

To clarify whether the reduction of resistance to antibiotics in the triple mutant is due to a defect in membrane composition or a lack of K^+^, the role of the single mutants was analyzed as well. Therefore, cells of all strains were prepared as described above and serial dilutions were dropped either on K^+^ free minimal medium containing 20 mM succinate and 8 μg/ml gentamicin (Fig. 8A) or on minimal medium containing 26.5 mM K^+^ and 8 μg/ml gentamicin as well (Fig. 8B). Again, the wild type was not affected by gentamicin, neither in the absence or presence of K^+^. In contrast, the triple mutant did not grow, neither in the absence or presence of K^+^. The ∆*trk* single mutant was unaffected by the presence of gentamicin, regardless of the availability of K^+^ in the assay. Furthermore, under K^+^ limiting conditions, growth of the ∆*kdp* deletion mutant was drastically reduced but growth was restored by the addition of K^+^. Growth of the ∆*kup* single and the double deletion mutant showed an adverse effect: at high K^+^ concentrations, growth was reduced drastically, but growth was restored at low K^+^ concentrations. This leads to the conclusion that K^+^ uptake mediated by Kup at high K^+^ concentrations and Kdp at low K^+^ concentrations is important for gentamicin resistance.

## Discussion

In previous studies, we demonstrated that the K^+^ transporter Kup, Trk, and Kdp of *A. baumannii* ATCC 19606 play a major role in the general adaptation to the human host, including amino acid utilization and infection of *Galleria mellonella* larvae (König et al. 2021). Here, we provide supporting information regarding its extended role in survival mechanisms required in the hospital environment.

The hospital is a harsh environment in which bacterial pathogens need to thrive to ensure proper transmission rates and successful infection of new hosts. One of the biggest challenges outside the human body is the severe nutrient limitation and non-favorable temperatures, e.g., room temperature. In the scenario we depicted here, the crucial role of a steady K^+^ supply and a stable K^+^ homeostasis becomes clear: cells of the triple mutant which were subjected to nutrient starvation at room temperature had a drastically reduced long-term survival rate in comparison to the wild type. The plethora of physiological processes that potassium ions are involved in gives rise to a broad range of plausible explanations for this phenotype (Epstein 2003; Beagle and Lockless 2021; Stautz et al. 2021). First, sufficient K^+^ availability is crucial for the proper function of many enzymes, including proteins involved in stress response and the ribosome itself (Epstein 2003). As described previously, the intracellular K^+^ level of the triple mutant is very low in comparison to the wild type, surely negatively influencing the activity of enzymes involved in general stress response or cold stress adaptation in particular (König et al. 2021). The importance of K^+^ in long-term survival was addressed in different organisms, for example, *Escherichia coli* and *Halobacterium salinarum* (Munro et al. 1989; Kixmüller and Greie 2012). Munro et al. (1989) pointed out that an efficient osmoadaptation process improved survival in seawater, while Kixmüller and Greie (2012) showed that the Kdp system is important for the survival of *H. salinarum* in seawater crystals. Although these results impede direct comparison with our data, these experiments underline the importance of efficient osmoadaptation in the process of long-term survival.

Maybe the most important role of a functional K^+^ homeostasis is its participation in membrane potential establishment and stabilization (Epstein 2003). For a living cell, the maintenance of a disequilibrium of charges across the membrane is crucial for generating and maintaining a proton motive force which then again is essential for ATP synthesis. During K^+^ import, protons are imported simultaneously. Deletion of major K^+^ uptake systems results in a higher electrochemical proton potential and thus ATP synthesis. This is what we indeed observed in the triple mutant. On the other hand, the wild type, like any other bacterial cell, accumulates K^+^ to high concentrations. In times of low energy supply, K^+^ can efflux the cells, driven by the K^+^ gradient (K^+^_in_ > K^+^_out_) and thus generate an electrical field (outside positive) that enables survival for some time in the absence of cellular respiration. Since the K^+^ transporter mutants no longer accumulate K^+^ at the first place, they cannot maintain a membrane potential by K^+^ leakage. This goes in line with the findings in *Corynebacterium glutamicum* and *Staphylococcus aureus* where the importance of bacterial K^+^ transporter in membrane potential regulation was already shown (Ochrombel et al. 2011; Gries et al. 2013, 2016).

Furthermore, we could show that the growth of the triple mutant in the presence of the antibiotics hygromycin B and gentamicin and the antimicrobial peptides colistin and polymyxin B was heavily impaired. This phenomenon has also been described for some bacteria including *S. aureus*. An intact membrane potential is involved in the protection against aminoglycoside entry into the cell (Mates et al. 1982). This goes in line with the findings of Gries et al. that the deletion of the Ktr K^+^ transport system resulted in reduced resistance against antibiotics and antimicrobials due to a hyperpolarized membrane (Gries et al. 2013, 2016).

Follow-up experiments with the single mutants and high doses of gentamicin enabled closer insights into the importance of the specific K^+^ transporter in antibiotic resistance. Interestingly, the only single mutant which did not grow under K^+^ limitation and antibiotic pressure was ∆*kdp*. The Kdp system is the only K^+^ transporter that is upregulated under K^+^ limitation and the main uptake system under these conditions. This leads to the assumption that ∆*trk* and ∆*kup* are not affected by gentamicin because the corresponding transporter are not active under these conditions. At high K^+^ concentrations, ∆*kdp* grew perfectly fine disproving the assumption of membrane instability causing the defect in antibiotic resistance. In contrast, ∆*kup* is highly defective against gentamicin under high K^+^ conditions. Since Kup is the main K^+^ uptake system under normal conditions (sufficient K^+^), this experiment proves its essentiality for antibiotic resistance under high K^+^ concentrations. Being constitutively expressed, Trk constitutes an exception since its deletion does not influence antibiotic resistance at all. This experiment proves that active K^+^ uptake is crucial for antibiotic resistance in *A. baumannii*. This finding makes Kup an interesting drug target.

## Conclusions

This study provides additional information regarding the importance of a stable K^+^ homeostasis for persistence in the nosocomial environment and antibiotic resistance in *A. baumannii* ATCC 19606. We could show that the disturbance of K^+^ import affects multiple adaptation mechanisms including metal stress and disinfectant resistance. Our findings emphasize the plethora of functions potassium ions are involved in and are giving further suggestions for the specific relevance of the secondary importer Kup in antibiotic resistance.

## Data Availability

All the data which was generated in this study is included in the manuscript and can be provided by the authors.
